# Supplementary material for: 3D Co-Printing and Substrate Geometry Influence the Differentiation of C2C12 Skeletal Myoblasts
Source: Gels. 2023 Jul 24;9(7):595. doi: 10.3390/gels9070595 (PMC10378771; doi:10.3390/gels9070595)
Supplement: Supplementary file 1 [file gels-09-00595-s001.zip › gels-2486740-supplementary.pdf]

Supporting information

Table S1. Summary of primers used for qRT-PCR.

Gene	Primer sequences
PGK (115 bp)	Fw 5’ CAA AAT GTC GTC TTC CAA CAA G 3’<br>Rw 5’AAC GTT GAA GTC CAC CCT CAT 3’
MyoD (115 bp)	Fw 5’ TGCACTTCCACCAACCCCAACCAGC 3’<br>Rw 5’ CCTGGACTCGCGCACCGCCTCACT 3’
Myf5 (176 bp)	Fw 5’ GCCCTGAGGAAGAGGAACAC 3’<br>Rw 5’ CAAGGTCTCGAATGCTTGGT 3’
Cyclin D1 (150 bp)	Fw 5’ TCCTGCTACCGCACAACGC 3’<br>Rw 5’ CCAGCTTCTTCCTCCACTTCCC 3’
MyoG (152 bp)	Fw 5’ GGGCCCCTGGAAGAAAAG 3’<br>Rw 5’ AGGAGGCGCTGTGGGAGTT 3’
MCK (103 bp)	Fw 5’ CCTGTTTGATCCCATCATCC 3’<br>Rw 5’ AGCACATAGTTGGGGTCCAG 3’
Myh1 (113 bp)	Fw 5’ AGTCCCAGGTCAACAAGCTG 3’<br>Rw 5’ CACATTTTGCTCATCTCTTTG 3’
MCad (...)	Fw5’ CTTGGGTGCCACGGATGA 3’<br>Rw 5’ATGCAGGCCCTCGGAGAC 3
